# PSYCHOSOCIAL FACTORS INFLUENCING DEPRESSIVE SYMPTOMS AMONG OLDER ADULTS FROM NORTHERN INDIA

**DOI:** 10.1093/geroni/igz038.1146

**Published:** 2019-11-08

**Authors:** Kasturi Banerjee, Tamara A Baker

**Affiliations:** University of Kansas, Lawrence, Kansas, United States

## Abstract

Global data show a significant increase in the number of adults 65+ years of age in India. Despite this increase, there is a dearth of available resources to adequately service their mental health needs. Data indicate that residents in Northern India, in particular, report poorer mental health outcomes than those in the South. The prevalence and impact of neuropsychiatric disorders and depression remain particularly significant, but largely unexplored. The aim of this study was to examine possible psychosocial and health factors affecting depressive symptoms in North India. Data were taken from the Longitudinal Ageing Study in India (LASI). Participants included adults aged 45 years and above (n=792), from the states of Rajasthan and Punjab. A multiple linear regression model was calculated to determine the influence of identified demographic and psychosocial factors (e.g., financial and social support, life satisfaction) on depressive symptoms. Data show that low life satisfaction (β= -0.19,p<0.001), poorer self-reported health (β=0.15,p<0.01), and being a care provider (β= -0.12,p<0.01) were significant predictors of depressive symptoms. These results indicate an increased need for care-giver mental health support along with policy aimed at awareness about caregiver burnout, health care access, and economic instrumental support services. A magnified view of the impact of life satisfaction on depression will be of immense value for understanding the unique needs and challenges of working with this population.

